# Bioactivities and Future Perspectives of Chaetoglobosins

**DOI:** 10.1155/2020/8574084

**Published:** 2020-03-24

**Authors:** Jinhua Chen, Wenzhou Zhang, Qingfeng Guo, Weijiang Yu, Yongna Zhang, Baoxia He

**Affiliations:** ^1^Department of Pharmacy, Affiliated Cancer Hospital of Zhengzhou University, Henan Cancer Hospital, Zhengzhou 450008, China; ^2^Zhengzhou Key Laboratory of Medicinal Resources Research, Huanghe Science and Technology College, Zhengzhou 450063, China

## Abstract

Chaetoglobosins belonging to cytochalasan alkaloids represent a large class of fungal secondary metabolites. To date, around 100 chaetoglobosins and their analogues have been isolated and identified over the years from a variety of fungi, mainly from the fungus *Chaetomium globosum*. Studies have found that chaetoglobosins possess a broad range of biological activities, including antitumor, antifungal, phytotoxic, fibrinolytic, antibacterial, nematicidal, anti-inflammatory, and anti-HIV activities. This review will comprehensively summarize the biological activities and mechanisms of action of nature-derived chaetoglobosins.

## 1. Introduction

**Figure 1:**
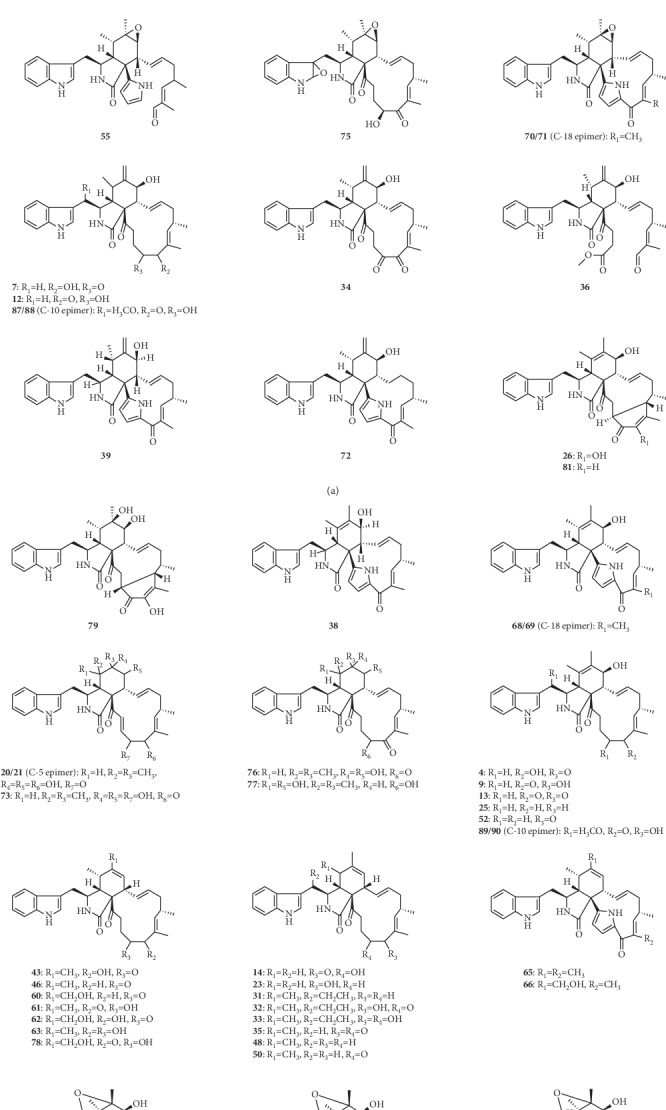
Chemical structures of chaetoglobosins.

Chaetoglobosins represent a large class of fungal secondary metabolites and belong to cytochalasan alkaloids, which contain a 10-(indol-3-yl) group, a macrocyclic ring, and a perhydroisoindolone moiety [1]. According to the chemical structure characteristics, they are divided into the subfamilies chaetoglobosin, penochalasin, prochaetoglobosin, armochaetoglasin, aureochaeglobosin, and oxichaetoglobosin (Figure 1). To date, around 100 chaetoglobosins and their analogues have been isolated and identified over the years from a variety of fungi, including *Chaetomium elatum* [2], *Chaetomium globosum* [3], *Phomopsis* sp. [4], *Botryosphaeria dothidea* [5], and *Chaetomium subaffine* [6], mainly from the fungus *Chaetomium globosum*.

Increasing evidence has indicated that chaetoglobosins possess a broad range of biological activities, including antitumor [2], antifungal [3], phytotoxic [7], fibrinolytic [7], antibacterial [8], nematicidal [9], anti-inflammatory [10], and anti-HIV activities [11] (Table 1). Therefore, they have broad application prospects and attract reseachers to further study. For better understanding and development of chaetoglobosins, we will review the biological activities and mechanisms of action of nature-derived chaetoglobosins.

## 2. Antitumor Activity

Cancer is the second leading cause of death throughout the world and is responsible for an estimated 9.6 million deaths in 2018. Studies have shown that lots of chaetoglobosins have potent antitumor activity in many types of tumor cell lines, such as HL60, A549, SMMC7721, and MCF-7 cell lines. There are three noteworthy characteristics of antitumor activity of chaetoglobosins: (1) chaetoglobosins had broad-spectrum antitumor activity. Compound **1** inhibited L929, KB3.1, PC-3, and HUVEC cell lines with the IC_50_ values of 1.6, 0.15, 0.42, and 0.78 *μ*g/mL, respectively [9]. Ruan et al. also demonstrated that compound **36** showed potent cytotoxicity to HL60, A549, SMMC7721, MCF-7, and SW480 cell lines with the range of inhibition ratio at 51–96% for a concentration of 40 *μ*mol/L [12]. In addition, compound **47** significantly inhibited growth of MDA-MB-435, SGC-7901, and A549 cell lines with IC_50_ values of 4.65, 5.32, and 8.73 *μ*mol/L, respectively [54]. (2) Different chaetoglobosins had similar inhibitory activity on the same tumor cell lines. Compounds **4** and **7** had showed significant growth inhibitory activity against BC1 cell lines with IC_50_ values of 3.03 *μ*mol/L and 7.2 *μ*mol/L, respectively, but both had no effect on cholangiocarcinoma cell lines (KKU-100 and KKU-OCA17) [2]. In study by Li et al., it also indicated that compounds **1**, **10**, **12**, **13**, **25**, and **26** exhibited antitumor activity against HCT116 cell line with IC50 values of 3.15, 17.8, 4.43, 65.6, 29.5, and 18.4 *μ*mol/L, respectively. Furthermore, the structure-activity analysis showed that the cytotoxicity was closely related with the epoxide ring at C-6-C-7 or a double bond at C-6 [12]. (3) Some of chaetoglobosins had differential actions on distinct subtype cell lines of the same tumor. The study by Thohinung et al. showed that compound **13** inhibited the growth of cholangiocarcinoma KKU-100 cell (IC_50_ = 29.85 *μ*mol/L), but had no inhibitory activity against the cholangiocarcinoma KKU-OCA17 cell line [2]. However, there are obvious disadvantages that are lack of animal experiments and the thorough study about structure-activity relationship. Therefore, further studies are needed to confirm the structure-activity relationship in order to better structural modification of lead compounds and obtain more effective drugs.

**Figure 2:**
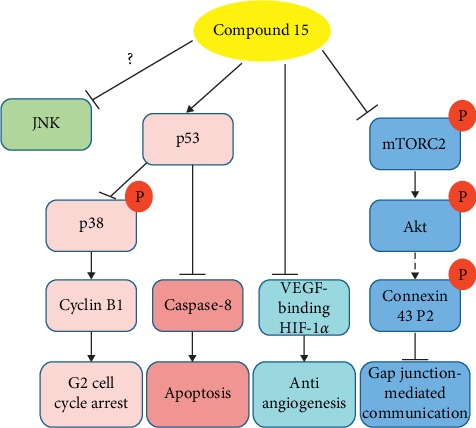
Antitumor mechanisms of action of compound **15**.

Currently, except for compound **15**, antitumor mechanisms of action of other chaetoglobosins were not reported. Studies have indicated that various mechanisms are involved in the antitumor activities of compound **15** (Figure 2). Ali and colleagues found that compound **15** supressed Ras-induced malignant phenotype due to its dual inhibitory effect on both Akt and JNK signaling pathways. Furthermore, Akt's two activation sites, T308 and S473, are known to be affected by treatment [54, 55]. Further study demonstrated that pretreatment with compound **15** decreased the phosphorylation at mTORC2 S2481, which phosphorylates Akt S473, comparable to Torin1, a known mTOR specific inhibitor. Therefore, it might be an mTOR inhibitor [33]. Moreover, administration of compound **15** to astroglial cell line can prevent and reverse the inhibition of lindane and dieldrin to gap junction-mediated communication, by stabilizing and reappearing the connexin 43 P2 phosphoform and activating the Akt/GSK-3*β* pathway [35–37]. Thus, we can infer that the mTOR/Akt/GSK-3*β* signaling pathway may play an important role in the antitumor action of compound **15**. Besides, Li et al. demonstrated that compound **15** showed a more potent cytotoxic to cisplatin-resistant ovarian cancer OVCAR-3 and A2780/CP70 cell lines than normal ovarian IOSE-364 cell line, by enhancing the p53-dependent caspase-8 activation extrinsic apoptosis pathway and inducing G2 cell cycle arrest via cyclin B1 by increasing p53 expression and p38 phosphorylation. However, it is needed to note that compound **15** did not have effects on phospho-JNK and total JNK in inhibition of growth of OVCAR-3 and A2780/CP70 cells, which was different from mechanism of action in Ras-transformed epithelial and human carcinoma cells through inhibition of the JNK signaling pathway [34, 55]. We inferred that its antitumor mechanisms of action might be tumor type-dependent, which need to get the experiment certification further. In addition, compound **15** can effectively inhibit angiogenesis through downregulation of VEGF-binding HIF-1 [38].

## 3. Antifungal Activity

Fungi are the principal causal agents of plant diseases. Several studies had revealed that chaetoglobosins exhibited significantly inhibitory activity against plant pathogenic fungi. For example, compound **1** displayed significant growth inhibitory activity against the fungi *Colletotrichum gloeosporioides* [1], *Fusarium sporotrichioides* [3], *Rhizopus stolonifer*, *Coniothyrium diplodiella* [18], *Setosphaeria turcica* [56], *Botrytis cinerea*, *Sclerotinia sclerotiorum* [57], and *Mucor miehei* [13]. In a study by Zhang et al., it reported that compounds **6**, **7**, **9**, **13**, and **21** inhibited *Rhizopus stolonifera* and *Coniothyrium diplodiella* [18]. Compounds **13**, **25**, and **26** have also been reported to inhibit *Alternaria solani* [31]. In addition, Huang et al. found that compounds **6**, **9**, **10**, **45**, **46**, and **63** displayed significant growth inhibitory activity against the fungi *Colletotrichum musae*, *Penicillium italicum Wehme*, *Rhizoctonia solani*, and *Colletotrichum gloeosporioides*. In comparison with other chaetoglobosins, compound **9** exhibited the highest antifungal activities. Based on the structure characteristics, we infer that C5-C6 double bond and C7-OH appear to greatly increase the antifungal potency [1]. Therefore, chaetoglobosins have a potential application value to control plant diseases.

## 4. Phytotoxic Activity

Chaetoglobosin exhibited significant inhibitory activity against many plant pathogenic fungi, indicating they might have a potential application value in agriculture. However, there are some literatures reported several chaetoglobosins showed phytotoxic activities. The study by Li et al. found that compounds **1**, **9**, **10**, **12**, **25** and **26** isolated from metabolites of *Stenocarpella maydis* showed remarkedly the growth inhibition of radish (*Raphanus sativus*) seedlings with inhibitory rates of >60% at a concentration of 50 ppm. The configurations of C-17 and C-21 in compounds **25** and **26** are closely related with phytotoxicity potency [12]. In addition, compounds **1**, **6** and **18** had also been reported inhibited the hypocotyl and root of Alfalfa seedings [27]. Therefore, the potential applications of chaetoglobosins in agriculture require comprehensive evaluation.

## 5. Antibacterial Effect

With antibacterial resistance becoming more and more serious, the search for new antibacterial agents is also urgent. Studies revealed that chaetoglobosins exhibited significant antibacterial activity against agricultural germs. Zhu et al. demonstrated that compound **17** is isolated from the solid culture of the mangrove endophytic fungus.*Penicillium chrysogenum* V11, possessed significantly antibacterial against *Colletotrichum gloeosporioides* with the IC_50_ value of 6.13 *μ*mol/L [49]. Except for inhibition against agricultural germs, it also showed the effective on clinical pathogenic bacteria. Hu and his colleague found that compound **57** showed antibacterial activity against *Klebsiella pneumoniae* (MIC = 4.0 *μ*g/mL) and *ESBL-producing Escherichia coli ATCC 35218* (MIC = 16.0 *μ*g/mL), wherein the inhibitory against *Klebsiella pneumoniae* was stronger than that of the clinically used antibiotic meropenem (MIC = 8 *μ*g/mL), [27]. Thus, these studies further indicated that they may have a great potential application value in agriculture and clinical aspects.

## 6. Immunomodulatory Property

**Figure 3:**
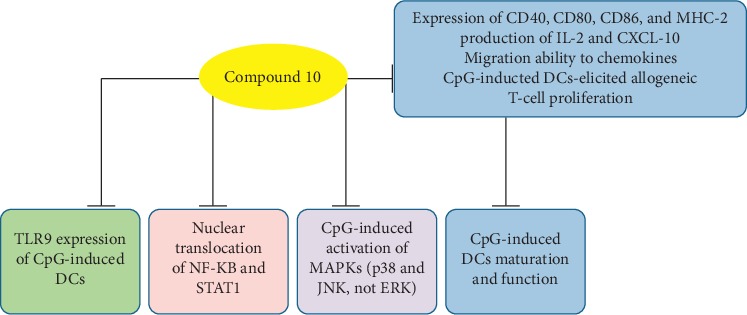
Mechanisms of action of immunomodulatory property of compound **10**.

Dendritic cells (DCs), the most potent antigen-presenting cells, possess both immune sentinels and initiators of T-cell response. It is the major target in the modulation of excessive immune responses. Hua et al. confirmed that compound **10** inhibited the CpG-induced DCs maturation and function and suppressed TLR9 expression of CpG-induced DCs through many signaling pathways. In addition, It also inhibited CpG-induced activation of MAPKs (p38 and JNK, but not ERK) and the nuclear translocation of NF-*κ*B and STAT1 (Figure 3) [29]. Therefore, compound **10** may have a great potential application in controlling DCs-associated autoimmune and/or inflammatory diseases.

## 7. Other Activities

In addition to the effects described above, studies showed that chaetoglobosins have some other activities, including fibrinolytic, anticoagulant, nematicidal, anti-HIV, and anti-inflammatory activities. Compound **1** was reported to inhibit J2 penetration and induce the production of urokinase in endothelial cells, associating with the elevation of fibrinolytic activity [7, 29]. Compound **36** showed antiacetylcholinesterase activity and weak anticoagulant activity with PT at 16.8 s [10]. Mori et al. also found compounds **1** and **51** showed antiamebic activities in the cysteine-deprived medium, in comparable to in the cysteine-containing medium [24]. In addition, compounds **66**, **67**, **68**, **71**, and **72** showed significant anti-HIV activities, with EC_50_ values ranging from 0.11 to 0.55 *μ*mol/L and selectivity index values ranging from 12.33 to 75.42 [11]. Compound **12** could inhibit NF-*κ*B and negatively regulated ERK1/2, p38, and JNK1/2 phosphorylations to exert anti-inflammatory property [30]. Therefore, chaetoglobosins have a great application prospect.

## 8. Conclusion

Microbial metabolites are important sources of discovery for drug lead compounds. The researchers extracted around 100 chaetoglobosins from the fungi's secondary metabolites and found that they possessed a broad range of biological activities, such as antitumor, antifungal, phytotoxic, and anti-HIV activities. Therefore, they attract reseachers to further study about antitumor and anti-microbial activities for better clinical application. However, it is needed to note that they have a dual role in agriculture, which is not only against plant-pathogenic fungi but also phytotoxic activities. Thus, the potential applications of chaetoglobosins in agriculture require comprehensive evaluation.

However, there are still some shortcomings in existing researches. Firstly, the research on chaetoglobosins remained *in vitro*, lack of *in vivo* animal experiments. Secondly, only a few chaetoglobosins have been elucidated about action mechanisms, but action mechanisms of most chaetoglobosins remained unclear. Thirdly, there was little research on the structure-activity relationship.

In conclusion, it is necessary to further evaluate their bioactivities *in vivo* experiments, their action mechanisms, and structure-activity relationship, thereby better and more comprehensive development and utilization of chaetoglobosins.

## Figures and Tables

**Table 1:** Summary of bioactive chaetoglobosins.

Number	Compounds	Activities	References
**1**	Chaetoglobosin A	Antitumor	[1, 9, 12–17]
		Antifungus	[1, 3, 18–20]
		Antibacterial	[8, 19, 21]
		Phytotoxicity	[12, 22]
		Nematicidal	[9, 23, 24]
		Fibrinolytic activity	[7]
**2**	19-O-Acetylchaetoglobosin A	Antitumor	[9]
		Nematicidal	[9]
**3**	20-Dihydrochaetoglobosin A	Antitumor	[12]
**4**	Chaetoglobosin B	Antitumor	[2, 14, 15, 18]
		Antifungus	[19]
		Antibacterial	[8, 19]
**5**	19-O-Acetylchaetoglobosin B	—	[25]
**6**	Chaetoglobosin C	Antitumor	[1, 2, 17, 26]
		Antifungal	[1, 19]
		Antibacterial	[27]
		Phytotoxicity	[12, 22]
**7**	Chaetoglobosin D	Antitumor	[2, 14, 15]
		Antifungal	[15, 18, 19]
		Antibacterial	[16]
**8**	19-O-Acetylchaetoglobosin D	—	[25]
**9**	Chaetoglobosin E	Antitumor	[1, 14, 16, 17, 26, 28]
		Antifungal	[1, 18]
		Phytotoxicity	[12]
**10**	Chaetoglobosin F	Antitumor	[1, 2, 12, 16, 17, 26]
		Phytotoxicity	[1, 12]
		Immunosuppressive property	[29]
**11**	Chaetoglobosin Fa	Antitumor	[12]
		Phytotoxicity	[12]
**12**	Chaetoglobosin F (ex)	Antitumor	[12, 17, 26]
		Phytotoxicity	[12]
		Antiinflammatory property	[30]
**13**	Chaetoglobosin G	Antitumor	[1, 2, 17, 18, 28]
		Antifungal	[1, 31]
		Antibacterial	[31]
**14**	Chaetoglobosin J	Antitumor	[15, 32]
**15**	Chaetoglobosin K	Antitumor	[18, 33–39]
		Antifungal	[40, 41]
**16**	Chaetoglobosin M	Antifungal	[4, 41]
**17**	Chaetoglobosin N	—	[4]
**18**	Chaetoglobosin O	Antitumor	[14, 42]
**19**	Chaetoglobosin P	—	[43]
**20**	Chaetoglobosin Q	Antitumor	[15]
**21**	Chaetoglobosin R	Antifungal	[18, 20]
**22**	Chaetoglobosin S	—	[31]
**23**	Chaetoglobosin T	Antitumor	[15]
		Antifungal	[20]
		Antibacterial	[20, 27]
**24**	Chaetoglobosin U	Antitumor	[16]
**25**	Chaetoglobosins V	Antitumor	[2, 12, 14, 17, 32, 44]
		Antifungal	[31]
		Antibacterial	[27, 31]
		Phytotoxicity	[12]
**26**	Chaetoglobosin V (b)	Antitumor	[12]
		Antifungal	[31]
		Antibacterial	[31]
		Phytotoxicity	[12]
**27**	Chaetoglobosin W	Antitumor	[17]
**28**	Chaetoglobosin X	Antitumor	[45]
		Antifungal	[45]
**29**	Chaetoglobosin Y	Antitumor	[28]
**30**	Chaetoglobosin Z	Antitumor	[14]
**31**	Chaetoglobosin-510	Antitumor	[46]
**32**	Chaetoglobosin-540	Antitumor	[46]
**33**	Chaetoglobosin-542	Antitumor	[46]
**34**	Isochaetoglobosin D	Antitumor	[2, 28]
**35**	Isochaetoglobosin J	—	[47]
**36**	Yamchaetoglobosin A	Antitumor	[10]
		Anticoagulant activity	[10]
**37**	Penochalasin A	Antitumor	[16, 48]
**38**	Penochalasin B	Antitumor	[48]
**39**	Penochalasin C	Antitumor	[26, 48]
**40**	Penochalasin D	Antitumor	[42]
**41**	Penochalasin E	Antitumor	[42]
**42**	Penochalasin F	Antitumor	[42]
**43**	Penochalasin G	Antitumor	[42]
**44**	Penochalasin H	Antitumor	[42]
**45**	Penochalasin I	Antitumor	[1]
		Antifungal	[1]
		Antibacterial	[27]
**46**	Penochalasin J	Antitumor	[1]
		Antifungal	[1]
**47**	Penochalasin K	Antitumor	[49]
		Antifungal	[49]
**48**	Prochaetoglobosin I	Antitumor	[15]
		Antibacterial	[27]
**49**	Isprochaetoglobosin I	—	[46]
**50**	Prochaetoglobosin II	Antitumor	[6, 15]
**51**	Prochaetoglobosin III	Antitumor	[2]
		Antiamebic	[24]
**52**	Prochaetoglobosin IIIed	Antitumor	[2]
**53**	Prochaetoglobosin IV	—	[47]
**54**	Trimethylated chaetoglobosin	—	[4]
**55**	Armochaetoglasins A	Antitumor	[50]
		Antibacterial	[27]
**56**	Armochaetoglasin B	Antitumor	[32, 51]
		Antibacterial	[27]
**57**	Armochaetoglasin C	Antitumor	[51]
		Antibacterial	[27]
**58**	Armochaetoglasin D	Antitumor	[51]
**59**	Armochaetoglasin E	Antitumor	[51]
**60**	Armochaetoglasin F	—	[51]
**61**	Armochaetoglasin G	Antitumor	[51]
**62**	Armochaetoglasin H	Antitumor	[51]
**63**	Armochaetoglasin I	Antitumor	[1, 51]
		Antifungal	[1]
**64**	Armochaetoglasin J	Antitumor	[51]
**65**	Armochaetoglasin K	Anti-HIV I	[11]
**66**	Armochaetoglasin L	Anti-HIV I	[11]
**67**	Armochaetoglasin M	Anti-HIV I	[11]
**68**	Armochaetoglasin N	Anti-HIV I	[11]
**69**	Armochaetoglasin O	Anti-HIV I	[11]
**70**	Armochaetoglasin P	Anti-HIV I	[11]
**71**	Armochaetoglasin Q	Anti-HIV I	[11]
**72**	Armochaetoglasin R	Anti-HIV I	[11]
**73**	Armochaetoglasin S	Antitumor	[50]
**74**	7-O-Acetylarmochaetoglobin S	Antitumor	[50]
**75**	Armochaetoglasin T	Antitumor	[50]
**76**	Armochaetoglasin U	Antitumor	[50]
**77**	Armochaetoglasin V	Antitumor	[50]
**78**	Armochaetoglasin W	Antitumor	[50]
**79**	Armochaetoglasin X	Antitumor	[50]
**80**	Armochaetoglasin Y	Antitumor	[50]
		Antibacterial	[27]
**81**	Armochaetoglasin Z	Antitumor	[50]
**82**	Aureochaeglobosin A	Antitumor	[52]
**83**	Aureochaeglobosin B	Antitumor	[52]
**84**	Aureochaeglobosin C	Antitumor	[52]
**85**	Oxichaetoglobosin A	Antitumor	[53]
		Immunomodulatory activity	[53]
**86**	Oxichaetoglobosin B	Antitumor	[53]
		Immunomodulatory activity	[53]
**87**	Oxichaetoglobosin C	Antitumor	[53]
		Immunomodulatory activity	[53]
**88**	Oxichaetoglobosin D	Antitumor	[53]
		Immunomodulatory activity	[53]
**89**	Oxichaetoglobosin E	Antitumor	[53]
		Immunomodulatory activity	[53]
**90**	Oxichaetoglobosin F	Antitumor	[53]
		Immunomodulatory activity	[53]
**91**	Oxichaetoglobosin G	Antitumor	[53]
		Immunomodulatory activity	[53]
**92**	Oxichaetoglobosin H	Antitumor	[53]
		Immunomodulatory activity	[53]
**93**	Oxichaetoglobosin I	Antitumor	[53]
		Immunomodulatory activity	[53]

## References

[1] Huang S., Chen H., Li W., Zhu X., Li C. (2016). Bioactive chaetoglobosins from the mangrove endophytic fungus *Penicillium chrysogenum*. *Marine Drugs*.

[2] Thohinung S., Kanokmedhakul S., Kanokmedhakul K., Kukongviriyapan V., Tusskorn O., Soytong K. (2010). Cytotoxic 10-(indol-3-yl)-[13]cytochalasans from the fungus *Chaetomium elatum* ChE01. *Archives of Pharmacal Research*.

[3] Jiang C., Song J., Zhang J., Yang Q. (2017). Identification and characterization of the major antifungal substance against *Fusarium Sporotrichioides* from *Chaetomium globosum*. *World Journal of Microbiology and Biotechnology*.

[4] Burlot L., Cherton J.-C., Convert O., Correia I., Dennetiere B. (2003). New chaetoglobosins from maize infested by *Phomopsis leptostromiformis* fungi. Production, identification, and semi‒synthesis. *Spectroscopy*.

[5] Xiao J., Zhang Q., Gao Y. Q., Tang J.-J., Zhang A.-L., Gao J.-M. (2014). Secondary metabolites from the endophytic *Botryosphaeria dothidea* of *Melia azedarach* and their antifungal, antibacterial, antioxidant, and cytotoxic activities. *Journal of Agricultural and Food Chemistry*.

[6] Oikawa H., Murakami Y., Ichihara A. (1993). 20-ketoreductase activity of chaetoglobosin a and prochaetoglobosins in a cell-free system of chaetomium subaffine and the isolation of new chaetoglobosins. *Bioscience, Biotechnology, and Biochemistry*.

[7] Shinohara C., Chikanishi T., Nakashima S. (2000). Enhancement of fibrinolytic activity of vascular endothelial cells by chaetoglobosin A, crinipellin B, geodin and triticone B. *The Journal of Antibiotics*.

[8] Flewelling A. J., Bishop A. L., Johnson J. A., Gray C. A. (2015). Polyketides from an endophytic *Aspergillus fumigatus* isolate inhibit the growth of *Mycobacterium tuberculosis* and MRSA. *Natural Product Communications*.

[9] Ashrafi S., Helaly S., Schroers H. J. (2017). *Ijuhya vitellina* sp. nov., a novel source for chaetoglobosin A, is a destructive parasite of the cereal cyst nematode *Heterodera filipjevi*. *PLoS One*.

[10] Ruan B.-H., Yu Z.-F., Yang X.-Q. (2018). New bioactive compounds from aquatic endophyte *Chaetomium globosum*. *Natural Product Research*.

[11] Chen C., Zhu H., Wang J. (2015). Armochaetoglobins K-R, anti-HIV pyrrole-based cytochalasans from *Chaetomium globosum* TW1-1. *European Journal of Organic Chemistry*.

[12] Li H., Xiao J., Gao Y.-Q., Tang J. J., Zhang A.-L., Gao J.-M. (2014). Chaetoglobosins from *Chaetomium globosum,* an endophytic fungus in Ginkgo biloba, and their phytotoxic and cytotoxic activities. *Journal of Agricultural and Food Chemistry*.

[13] Knudsen P. B., Hanna B., Ohl S. (2014). Chaetoglobosin A preferentially induces apoptosis in chronic lymphocytic leukemia cells by targeting the cytoskeleton. *Leukemia*.

[14] Jiang T., Wang M., Li L. (2016). Overexpression of the global regulator LaeA in *Chaetomium globosum* leads to the biosynthesis of chaetoglobosin Z. *Journal of Natural Products*.

[15] Jiao W., Feng Y., Blunt J. W., Cole A. L. J., Munro M. H. G. (2004). Chaetoglobosins Q, R, and T, three further new metabolites fromChaetomiumglobosum. *Journal of Natural Products*.

[16] Munro G., Song Y. C., Chen J. R. (2006). Chaetoglobosin U, a cytochalasan alkaloid from endophytic *Chaetomium globosum* IFB-E019. *Journal of Natural Products*.

[17] Zhang J., Ge H., Jiao R. (2010). Cytotoxic chaetoglobosins from the endophyte *Chaetomium globosum*. *Planta Medica*.

[18] Zhang G., Zhang Y., Qin J. (2013). Antifungal metabolites produced by *Chaetomium globosum* no. 4, an endophytic fungus isolated from *Ginkgo biloba*. *Indian Journal of Microbiology*.

[19] Zhao S. S., Zhang Y. Y., Yan W., Cao L.-L., Xiao Y., Ye Y.-H. (2017). *Chaetomium globosum* CDW7, a potential biological control strain and its antifungal metabolites. *FEMS Microbiology Letters*.

[20] Yan W., Cao L.-L., Zhang Y.-Y. (2018). New metabolites from endophytic fungus *Chaetomium globosum* CDW7. *Molecules*.

[21] Dissanayake R. K., Ratnaweera P. B., Williams D. E. (2016). Antimicrobial activities of endophytic fungi of the Sri Lankan aquatic plant *Nymphaea nouchali* and chaetoglobosin A and C, produced by the endophytic fungus *Chaetomium globosum*. *Mycology*.

[22] Ichihara A., Katayama K., Teshima H., Oikawa H., Sakamura S. (1996). Chaetoglobosin O and other phytotoxic metabolites from *Cylindrocladium floridanum*, a causal fungus of alfalfa black rot disease. *Bioscience, Biotechnology, and Biochemistry*.

[23] Hu Y., Zhang W., Zhang P., Ruan W., Zhu X. (2012). Nematicidal activity of chaetoglobosin A poduced by *Chaetomium globosum* NK102 against *Meloidogyne incognita*. *Journal of Agricultural and Food Chemistry*.

[24] Mori M., Shiomi K., Nozaki T. (2018). Discovery of antiamebic compounds that inhibit cysteine synthase from the enteric parasitic protist *Entamoeba histolytica* by screening of microbial secondary metabolites. *Frontiers in Cellular and Infection Microbiology*.

[25] Probst A., Tamm C. (1981). 19-O-acetylchaetoglobosin B and 19-O-acetylchaetoglobosin D, two new metabolites ofChaetomium globosum. *Helvetica Chimica Acta*.

[26] Shen L., Zhu L., Wei Z. Q., Li X. W., Li M., Song Y. C. (2015). Chemical constituents from endophyte *Chaetomium globosum* in Imperata cylindrical. *Zhongguo Zhong Yao Za Zhi*.

[27] Gao W., He Y., Li F. (2019). Antibacterial activity against drug-resistant microbial pathogens of cytochalasan alkaloids from the arthropod-associated fungus *Chaetomium globosum* TW1-1. *Bioorganic Chemistry*.

[28] Zheng Q.-C., Kong M.-Z., Zhao Q. (2014). Chaetoglobosin Y, a new cytochalasan from *Chaetomium globosum*. *Fitoterapia*.

[29] Hua C., Yang Y., Sun L., Dou H., Tan R., Hou Y. (2013). Chaetoglobosin F, a small molecule compound, possesses immunomodulatory properties on bone marrow-derived dendritic cells via TLR9 signaling pathway. *Immunobiology*.

[30] Dou H., Song Y., Liu X. (2011). Chaetoglobosin Fex from the marine-derived endophytic fungus inhibits induction of inflammatory mediators via toll-like receptor 4 signaling in macrophages. *Biological & Pharmaceutical Bulletin*.

[31] Xue M., Zhang Q., Gao J.-M., Li H., Tian J.-M., Pescitelli G. (2012). Chaetoglobosin Vb from endophytic *Chaetomium globosum*: absolute configuration of chaetoglobosins. *Chirality*.

[32] Gao W., Sun W., Li F. (2018). “Armochaetoglasins A–I: cytochalasan alkaloids from fermentation broth of *Chaetomium globosum* TW1-1 by feeding L-tyrosine. *Phytochemistry*.

[33] Curless B. P., Uko N. E., Matesic D. F. (2019). Modulator of the PI3K/Akt oncogenic pathway affects mTOR complex 2 in human adenocarcinoma cells. *Investigational New Drugs*.

[34] Li B., Gao Y., Rankin G. O. (2015). Chaetoglobosin K induces apoptosis and G2 cell cycle arrest through p53-dependent pathway in cisplatin-resistant ovarian cancer cells. *Cancer Letters*.

[35] Matesic D. F., Blommel M. L., Sunman J. A., Cutler S. J., Cutler H. G. (2001). Prevention of organochlorine-induced inhibition of gap junctional communication by chaetoglobosin K in astrocytes. *Cell Biology and Toxicology*.

[36] Sidorova T. S., Matesic D. F. (2008). Protective effect of the natural product, Chaetoglobosin K, on lindane-and dieldrin-induced changes in astroglia: identification of activated signaling pathways. *Pharmaceutical Research*.

[37] Matesic D. F., Ali A., Sidorova T. S., Burns T. J. (2013). A cell-cell communication marker for identifying targeted tumor therapies. *Current Bioactive Compounds*.

[38] Luo H., Li B., Li Z., Cutler S. J., Rankin G. O., Chen Y. C. (2013). Chaetoglobosin K inhibits tumor angiogenesis through downregulation of vascular epithelial growth factor-binding hypoxia-inducible factor 1*α*. *Anti-Cancer Drugs*.

[39] Matesic D. F., Villio K. N., Folse S. L., Garcia E. L., Culter S. J., Culter H. G. (2006). Inhibition of cytokinesis and akt phosphorylation by chaetoglobosin K in ras-transformed epithelial cells. *Cancer Chemotherapy and Pharmacology*.

[40] Wicklow D. T., Rogers K. D., Dowd P. F., Gloer J. B. (2011). Bioactive metabolites from *Stenocarpella maydis*, a stalk and ear rot pathogen of maize. *Fungal Biology*.

[41] Rogers K. D., Cannistra J. C., Gloer J. B., Wicklow D. T. (2014). Diplodiatoxin, chaetoglobosins, and diplonine associated with a field outbreak of stenocarpella ear rot in Illinois. *Mycotoxin Research*.

[42] Iwamoto C., Yamada T., Ito Y., Minoura K., Numata A. (2001). Cytotoxic cytochalasans from a *Penicillium* species separated from a marine alga. *Tetrahedron*.

[43] Donoso R., Rivera-Sagredo A., Hueso-Rodríguez J. A., Elson S. W. (1997). A new chaetoglobosin isolated from a fungus of the genus *discosia*. *Natural Product Letters*.

[44] Elson D., Chen X., Zhu L. (2017). Chemical constituents of liquid culture of symbiotic *Chaetomium globosum* ML-4 of oyster and their *in vitro* antitumor activity. *Zhong Guo Zhong Yao Za Zhi*.

[45] Wang Y., Xu L., Ren W., Zhao D., Zhu Y., Wu X. (2012). Bioactive metabolites from *Chaetomium globosum* L18, an endophytic fungus in the medicinal plant Curcuma wenyujin. *Phytomedicine*.

[46] Christian O. E., Compton J., Christian K. R., Mooberry S. L., Mooberry F. A., Crews P. (2005). Using jasplakinolide to turn on pathways that enable the isolation of new chaetoglobosins from *Phomospis asparagi*. *Journal of Natural Products*.

[47] Oikawa H., Murakami Y., Ichihara A. (1992). Useful approach to find the plausible biosynthetic precursors of secondary metabolites using P-450 inhibitors: postulated intermediates of chaetoglobosin A. *Journal of the Chemical Society, Perkin Transactions 1*.

[48] Numata A., Takahashi C., Ito Y. (1996). Penochalasins, a novel class of cytotoxic cytochalasans from a *Penicillium* species separated from a marine alga: structure determination and solution conformation. *Journal of the Chemical Society, Perkin Transactions 1*.

[49] Zhu X., Zhou D., Liang F., Wu Z., She Z., Li C. (2017). Penochalasin K, a new unusual chaetoglobosin from the mangrove endophytic fungus *Penicillium chrysogenum* V11 and its effective semi-synthesis. *Fitoterapia*.

[50] Chen C., Tong Q., Zhu H. (2016). Nine new cytochalasan alkaloids from *Chaetomium globosum* TW1-1 (Ascomycota, Sordariales). *Scientific Reports*.

[51] Chen C., Wang J., Liu J. (2015). Armochaetoglobins A-J: cytochalasan alkaloids from Chaetomium globosum TW1-1, a fungus derived from the terrestrial arthropod *Armadillidium vulgare*. *Journal of Natural Products*.

[52] Yang M.-H., Gu M.-L., Han C. (2018). Aureochaeglobosins A-C, three [4 + 2] adducts of chaetoglobosin and aureonitol derivatives from *Chaetomium globosum*. *Organic Letters*.

[53] Wang W., Gong J., Liu X. (2018). Cytochalasans produced by the coculture of *Aspergillus flavipes* and *Chaetomium globosum*. *Journal of Natural Products*.

[54] Tikoo A., Cutler H., Lo S. H., Chen L. B., Maruta H. (1999). Treatment of Ras-induced cancers by the F-actin cappers tensin and chaetoglobosin K, in combination with the caspase-1 inhibitor N1445. *The Cancer Journal From Scientific American*.

[55] Ali A., Sidorova T. S., Matesic D. F. (2013). Dual modulation of JNK and Akt signaling pathways by chaetoglobosin K in human lung carcinoma and ras-transformed epithelial cells. *Investigational New Drugs*.

[56] Zhang G., Wang F., Qin J. (2013). Efficacy assessment of antifungal metabolites from *Chaetomium globosum* No. 05, a new biocontrol agent, against Setosphaeria turcica. *Biological Control*.

[57] Zhang W. H., Guo Z., Wei S. P., Ji Z. (2014). Investigation on the antimicrobial ingredients of *Chaetomium globosum* ZH-32, an endophytic fungus from platycladus orientalis. *Chinese Journal of Pesticide Science*.

